# Pseudo-professional mechanism of collaborative mental health support in low-resource county-level contexts in China

**DOI:** 10.3389/fpsyt.2026.1930270

**Published:** 2026-09-07

**Authors:** Yan Wang, Dan Chen

**Affiliations:** 1 School of Public Policy and Management, China University of Mining and Technology, Xuzhou, China; 2 School of Sociology, Beijing Normal University, Beijing, China; 3 School of Food Science and Bioengineering, Xuzhou University of Technology, Xuzhou, Jiangsu, China

**Keywords:** adolescent mental health, county-level mental health services, health psychology, pseudo-professional mechanism, relational embeddedness, school-family-community collaboration

## Abstract

China has made school-family-community collaboration a central policy direction for adolescent mental health, yet low-resource counties often lack the professional workforce, stable interorganizational arrangements, and non-stigmatizing pathways needed to reproduce an institutionalized service model. Existing research on task sharing shows that trained non-specialists can perform selected forms of basic psychological support, but it says less about how such tasks are incorporated into everyday school relationships and linked to professional referral. This community case study examines Suining County, Jiangsu Province, as an information-rich instrumental case. The core evidence consists of semi-structured interviews with 52 principals, school mental health teachers, homeroom teachers, subject teachers, parents, and students, together with non-participant observations conducted in 10 schools between September 2023 and June 2024. County policy documents, training records, and de-identified aggregate administrative screening data were used only to describe the case context. The analysis identifies a pseudo-professional mechanism organized through three related processes. Daily relational embeddedness enables risk signals to be noticed in classrooms, class management, and home-school communication. Bounded task sharing converts listening, basic reassurance, risk inquiry, and referral into limited front-end tasks that existing school actors can perform without assuming diagnostic or therapeutic authority. Tiered networking transfers responsibility from the class to the school and, when necessary, to medical services, while reconnecting students to everyday support after their return to school. This arrangement widens the reach of front-end support but remains constrained by teacher workload, parental refusal, discontinuities across school transitions, uneven supervision, and privacy risks. Pseudo-professionalization therefore does not mean replacing clinical professionals. It denotes a bounded reorganization of support functions whose safety depends on professional gatekeeping, sustained supervision, stable referral channels, informed participation, and disciplined information governance.

## 1 Introduction

Childhood and adolescence are critical periods for mental health development and for the onset of many mental disorders. Mental disorders account for a substantial share of the disease burden among young people, while low-resource settings face particularly pronounced gaps in professional staffing, service access, and continuity of care (1, 2). National surveys and systematic research in China likewise indicate a considerable burden of depression, anxiety, and other mental health problems among children and adolescents. Family migration, educational pressure, and limited access to professional services place some rural and county-level students at heightened risk (3–6).

China’s national Action Plan for Strengthening and Improving Student Mental Health in the New Era (2023–2025) calls for schools, families, communities, and public agencies to participate in promotion, monitoring, counseling, and crisis response (7). Epstein’s theory of overlapping spheres of influence likewise treats student development as the product of intersecting school, family, and community environments (8).

Policy commitments, however, do not automatically generate local service capacity. Chinese studies have found that home-school cooperation often remains one-directional, with parents positioned mainly as recipients of information or as implementers of school requests. Links between schools, medical services, and community organizations may also remain formalistic or fragmented because responsibilities, information-sharing rules, and referral resources are not securely established (9–11). These tensions are especially visible at the county level. Local authorities can launch screening programs, training initiatives, and special campaigns relatively quickly, whereas professional workforce development, clinical capacity, and routine cross-sector coordination require longer periods of institution-building.

Task shifting and task sharing have become important responses to mental health service gaps. These approaches assign selected structured, lower-threshold tasks to trained and supervised non-specialists in order to extend the reach of services for common mental health problems (12, 13). Most studies concern community health workers or project-based lay providers. Less is known about how front-end tasks enter existing school roles and connect to professional referral.

The present study addresses two related questions. How does adolescent mental health support enter students’ everyday lives in a county where full-time professional services are limited and a complete school-family-community collaborative system has not yet formed? How are role boundaries, responsibility transfer, and risk control maintained when non-specialists undertake basic support tasks? Suining County in Jiangsu Province is treated as an information-rich instrumental case rather than as a statistically representative model of all Chinese counties.

The study uses the term pseudo-professional mechanism as an analytical concept. The term is not a judgment about the status of participating actors, nor does it imply that non-professionals may diagnose or treat mental disorders. It refers to an organizational arrangement in which selected front-end and peripheral functions of a professional service chain are decomposed, taught, and procedurally incorporated into the everyday work of homeroom teachers, subject teachers, parents, and other existing actors. The analysis therefore asks not merely whether non-professionals participate, but which tasks they undertake, how those tasks are embedded in social relations, and where professional authority is retained.

## 2 Theoretical background and analytical framework

### 2.1 Service gaps and bounded task sharing

Task sharing begins by separating complex professional services into tasks with different knowledge requirements and levels of risk. It then specifies which tasks may be performed by trained non-specialists and which must remain with appropriately qualified professionals. The MANAS trial made this logic explicit: screening, psychoeducation, supportive listening, and selected brief interventions could be delivered by trained lay counselors, whereas diagnosis, medication, and complex treatment remained within the professional system (12). Evidence from low- and middle-income settings similarly emphasizes identifying tasks that can be reassigned safely under standardized protocols, ongoing supervision, and clear referral conditions (13). The World Health Organization’s mhGAP approach matches service complexity and risk to different provider levels (14).

School-based task sharing has a distinctive relational setting. Homeroom and subject teachers are not temporary project workers. They interact with students repeatedly, hold educational responsibilities, and observe behavior across classrooms, meals, recess, and home-school communication. This proximity may make changes in mood or participation more visible and may reduce the stigma associated with entering a counseling room. Yet the same relationship contains evaluative and disciplinary power. Students may hesitate to disclose distress to teachers who influence grades, disciplinary records, and communication with parents. Existing relationships may create trust, but they may also create caution. Confidentiality rules, explicit role descriptions, and reliable professional referral are therefore not secondary protections; they are conditions under which school-based task sharing can operate safely.

### 2.2 Relational embeddedness and the everyday accessibility of support

Embeddedness theory holds that action is shaped by concrete and continuing social relations rather than occurring between isolated individuals or within abstract institutional designs (15). The accessibility of mental health support likewise depends on more than the number of formal service sites. Risk signals must be recognizable in familiar interactions, students must be able to begin seeking help through relationships they regard as trustworthy, and professional resources must be reachable through the networks surrounding everyday life. Daily relational embeddedness, as used here, refers to placing ordinary observation, supportive listening, information reporting, and return-to-school support within classrooms, class management, peer interaction, and home-school communication.

Daily embeddedness should not be confused with the psychiatric labeling of ordinary life. Temporary sadness, academic frustration, and peer conflict should not be casually treated as signs of disorder. The defensible sequence is low-threshold detection, limited response, and timely referral—not the expansion of teachers’ authority to make informal diagnoses. Relational networks become support networks only when the scope of observation, access to information, reporting routes, and professional boundaries are regulated.

### 2.3 Three analytical dimensions of the pseudo-professional mechanism

The framework distinguishes three related dimensions. Daily relational embeddedness describes how basic support functions enter sustained interactions among teachers, parents, and peers. Functional diffusion, expressed empirically as bounded task sharing, describes how selected front-end functions are decomposed and converted into skills that non-specialists can understand and perform. Tiered networking describes how responsibility and information move to school mental health teachers and medical services when a problem exceeds the competence of the current actor. The three dimensions operate across four work stages: risk identification, initial response, professional referral, and return-to-school support (**Table 1**).

**Table 1:** Core concepts, empirical indicators, and risk boundaries.

Analytical dimension	Operational definition	Primary actors	Empirical indicators	Main risks and guardrails	Theoretical basis
Daily relational embeddedness	Placing ordinary observation, supportive communication, and ongoing support within classrooms, class management, and home-school communication	Homeroom teachers, subject teachers, parents, and student peers	Daily observation; unusual signals in classwork; two-way home-school feedback; continuing contact after return	Over-monitoring, stigma, and privacy breaches; limit the scope of records and access permissions	Granovetter (15): embeddedness
Functional diffusion (bounded task sharing)	Converting listening, basic reassurance, risk inquiry, and resource linkage into trainable basic tasks	Homeroom teachers, subject teachers, and parent volunteers; school mental health teachers provide support and supervision	Training content; communication scripts; risk red lines; referral triggers	Unauthorized diagnosis, inappropriate reassurance, and teacher burden; provide ongoing supervision and specify what actors must not do	Patel et al. (12)Singla et al. (13): task sharing and functional decomposition; WHO (14): mhGAP
Tiered networking	Creating responsibility transfer, professional intake, and information feedback among class, school, and medical levels	Homeroom teachers, school mental health teachers, administrators, medical services, and guardians	Tier criteria; referral procedures; response timeframes; case management; return-to-school support	Parental refusal, absence of a receiving service, and information discontinuity; require informed participation, stable pathways, and responsibility tracking	Bower and Gilbody (16): stepped care; WHO (14): tiered referral

## 3 Case context and methods

### 3.1 Case context

Suining County is located in northwestern Jiangsu Province and covers 1,769 km² (17). The county had a permanent population of 1.0665 million and an urbanization rate of 59.64% in 2024 (18). Local education records report 110 primary and secondary schools serving approximately 140,000 students. Labor migration, parent-child separation, and grandparental care form part of the social environment in which some local students grow up. Previous research has shown that parental migration may affect children’s psychosocial well-being through reduced parent-child interaction, changes in caregiving arrangements, and weaker emotional support (5, 6).

The term ‘low resource’ refers here to constraints in adolescent mental health support rather than to the county’s economy as a whole. During the study period, the county employed 79 full-time school mental health teachers, and only about 35% of schools had a full-time post. Another 285 teachers held intermediate-level or higher relevant certificates, mostly for part-time work. Since 2023, the county has allocated RMB 800,000 annually to screening and counseling interventions and established a guidance center, teacher training, and school-hospital referral initiatives. Policy mobilization was substantial, but staffing, professional development, and routine cross-sector coordination remained incomplete.

Earlier local materials used a teacher-student ratio to estimate a shortage of more than 30 full-time mental health teachers. Because policy documents differed in whether the ratio referred to full-time or part-time provision, the present study does not retain that estimate. It reports only verifiable staffing, school coverage, and student numbers. In the 2024 platform report, 4.78% of primary-school records and 9.91% of secondary-school records were flagged as high risk. Since complete psychometric documentation for the platform could not be independently verified, these figures are used only as contextual indicators of the scale of screening demand (**Table 2**).

**Table 2:** Main contextual indicators of student mental health support in Suining County.

Indicator	Value	Source	Use in this study
Permanent population and urbanization rate	1.0665 million; 59.64%	General Office of Suining County Government (18)	County demographic context
Primary and secondary schools and enrolled students	110 schools; approximately 140,000 students	Suining County Education Bureau (19)	Scale of service demand
Full-time school mental health teachers	79; about 35% of schools had a full-time post	Suining County Education Bureau (19)	Professional coverage and uneven school distribution
Teachers with intermediate-level or higher relevant certification	285; most worked part time in mental health support	Suining County Education Bureau (19)	Potential workforce for bounded task sharing
County-level earmarked expenditure	RMB 800,000 annually since 2023	Suining County Education Bureau (19)	Degree of policy mobilization
Records flagged high risk by the platform in 2024	Primary schools: 4.78%; secondary schools: 9.91%	Suining County Education Bureau (20)	Context only; not treated as clinical prevalence or a mechanism-effect indicator

### 3.2 Research design and case selection

The study adopts a qualitatively led community case study design. Case studies are well suited to questions about how mechanisms operate and how institutional arrangements are enacted in real settings (21). Suining was selected not because it statistically represents all counties, but because professional service constraints, strong policy mobilization, and sustained school-based initiatives coexist there. This combination makes the county an information-rich setting for examining the formation of a supplementary support mechanism. Information-rich single cases can identify mechanisms, refine concepts, and specify conditions for subsequent comparison, but they do not support unbounded generalization (22).

The county administrative screening data serve only a contextual purpose. The core evidence comes from interviews, observation, and documentary materials. The study is therefore not presented as a convergent mixed-methods design, and the screening data are not used to test the effectiveness of the pseudo-professional mechanism.

### 3.3 Data sources and participants

Qualitative data were collected from September 2023 to June 2024 using purposive and snowball sampling. The sample included 52 distinct participants: six principals, 10 school mental health teachers, 14 homeroom teachers, eight subject teachers, eight parents, and six students. Adult interviews lasted 45–90 min; student interviews lasted approximately 20–30 min. Interviews were audio-recorded and transcribed with participants’ consent. Participant characteristics and inclusion criteria are summarized in **Table 3**.

**Table 3:** Interview participants and main inclusion criteria.

Participant group	n	Gender	Age/experience/grade	School types represented	Main inclusion criterion
Principals	6	5 men, 1 woman	Age 42–55; 18–32 years of teaching experience	2 urban senior secondary; 1 urban junior secondary; 1 township junior secondary; 1 township primary	Direct responsibility for school mental health management or resource coordination
School mental health teachers	10	3 men, 7 women	Age 27–48; 4–22 years of teaching experience	2 urban senior secondary; 2 urban junior secondary; 3 township junior secondary; 1 township primary	Responsible for mental health classes, risk assessment, counseling, or referral
Homeroom teachers	14	5 men, 9 women	Age 28–53; 6–30 years of teaching experience	2 urban senior secondary; 2 urban junior secondary; 3 township junior secondary; 1 township primary	Experience in daily observation, student conversations, or risk reporting
Subject teachers	8	3 men, 5 women	Age 25–49; 2–26 years of teaching experience	1 urban senior secondary; 1 urban junior secondary; 2 township junior secondary; 1 township primary	Participation in subject-integrated mental health education or identification of unusual classroom signals
Parents	8	2 men, 6 women	Age 30–49	1 urban junior secondary; 1 township junior secondary; 1 township primary	Experience communicating with the school or accompanying a student to medical care or return to school
Students	6	3 boys, 3 girls	Age 10–17; Grade 4 to Grade 11	1 urban junior secondary; 1 township junior secondary; 1 township primary	Guardian consent and the student’s voluntary assent

Numbers in the school-type column indicate the number of schools represented, not the number of participants. More than one participant could come from the same school. The rural teaching site was included in observation but not in the interview sample. Role-based codes were used for data management and quotation tracing.

Non-participant observation was conducted in two phases across 10 schools. Nine of the schools also contributed interview participants; the rural teaching site contributed observation data only. Five schools were visited between November and December 2023 and five in May 2024. The sites comprised two urban senior secondary schools, two urban junior secondary schools, three township junior secondary schools, two township primary schools, and one rural teaching site. Each visit lasted approximately 3–4 h and covered mental health classes, counseling-room operations, recess and lunch interactions, and parent meetings. Five members of the research team, including the first author, participated in the observations. Teams of two or three prepared separate fieldnotes after each visit and compared them on the same day.

The county-level student mental health screening records were pre-existing secondary administrative data. Their use was authorized by the Suining County Education Bureau, and the research team received only de-identified aggregate statistics. The data were used to describe the case and were not used for individual diagnosis, prevalence estimation, causal inference, or evaluation of mechanism effectiveness. All adult interviewees provided written informed consent after receiving information about the study purpose, interview content, intended use of the materials, and their right to withdraw. Students participated only after guardian consent and their own voluntary assent. Recordings, transcripts, and fieldnotes were anonymized, with role-based codes replacing names, school identities, and other identifying information. Access was restricted to the research team.

The secondary aggregate data came from the county-wide 2024 mental health screening. A third-party technology company developed the platform, and the county report stated that the assessment framework drew on the Mini International Neuropsychiatric Interview (M.I.N.I. 7.0). The platform assigned records to five risk levels: levels 1–2 low risk, level 3 medium risk, and levels 4–5 high risk. Of 142,166 completed assessments, the platform retained 139,993 records as valid, a valid-response rate of 98.47%. The research team did not obtain independently verifiable documentation on the instrument’s items, scoring, reliability, validity, age applicability, or invalid-response rules. Platform flags are therefore not interpreted as diagnoses or prevalence estimates. **Table 4** reports the risk-flag categories as they appeared in the administrative report.

**Table 4:** Risk-flag rules used by the administrative screening platform.

Platform assessment signal	Risk level	Platform label	Risk category
No clear psychological risk signal	Level 1	Green	Low risk
Low mood without a medium- or high-risk flag	Level 2	Blue	Low risk
A risk signal related to panic, generalized anxiety, or social anxiety	Level 3	Yellow	Medium risk
A depression-related risk signal	Level 4	Orange	High risk
A depression-related risk signal accompanied by strong suicidal ideation	Level 5	Red	High risk

Labels reproduce the categories used in the administrative platform report. Levels 1–2 were classified as low risk, level 3 as medium risk, and levels 4–5 as high risk. The study does not treat these labels as clinical diagnoses or prevalence measures.

### 3.4 Data analysis

Interview transcripts, fieldnotes, and documentary materials were analyzed using reflexive thematic analysis (23). Two researchers familiarized themselves with the materials and developed initial codes. Constant comparison, analytic memos, and team discussion were used to reflect on differences in interpretation rather than to calculate inter-coder reliability. Initial process codes asked who noticed a problem, which task was undertaken, when escalation occurred, who received responsibility, and how support continued after a student’s return. The analysis then developed three themes: daily relational embeddedness, bounded task sharing, and tiered networking. Attention was also directed to materials that did not fit a successful-mechanism narrative, including parental refusal, teacher burden, information discontinuity, and privacy concerns. NVivo 14 supported organization and retrieval but did not replace interpretive judgment.

## 4 Findings

**Figure 1:**
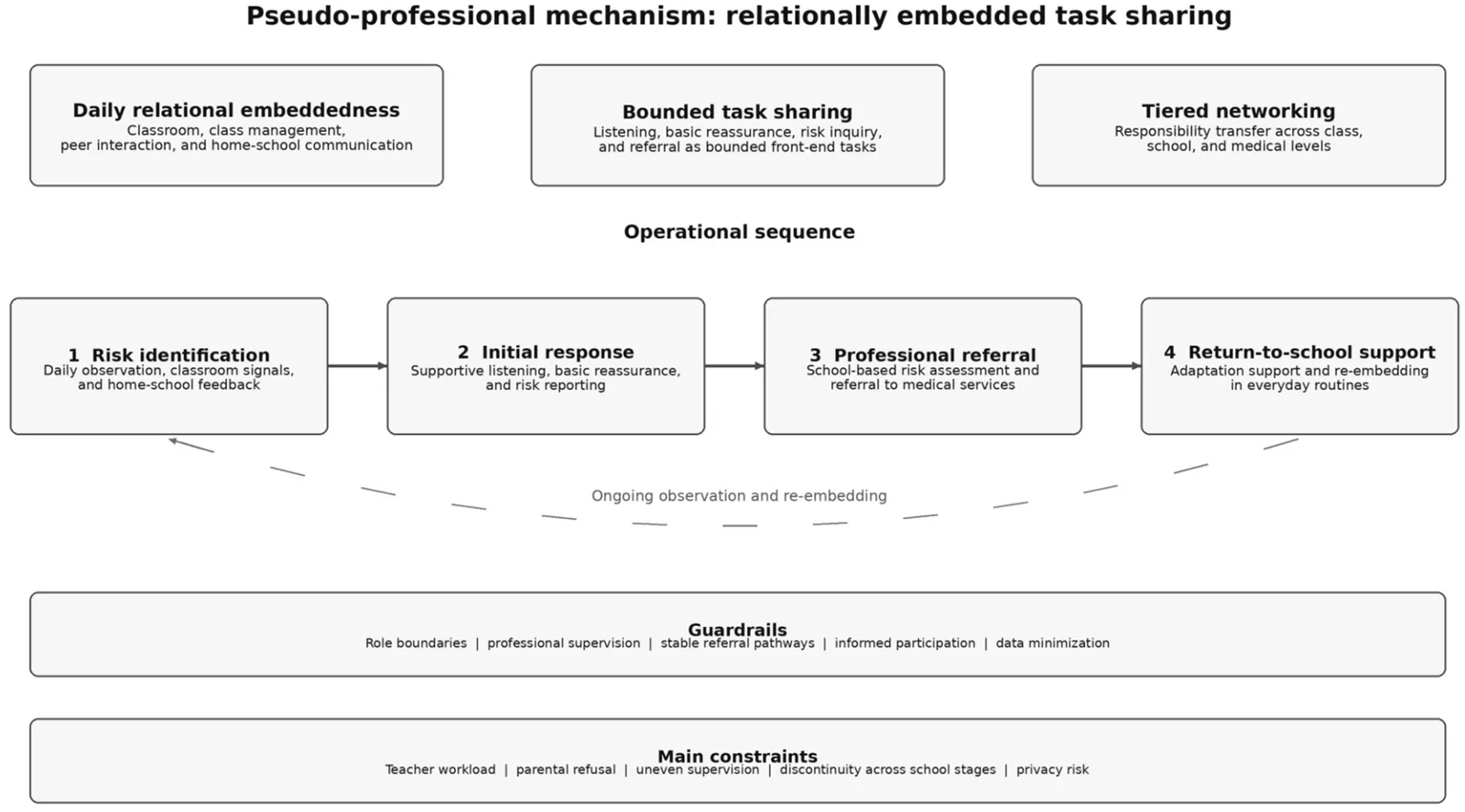
Structure and operational sequence of the pseudo-professional mechanism. Source: developed by the authors from interviews, field observations, county project documents, and school records.

The evidence indicates that Suining had not established a stable and comprehensive system linking education, medical services, community organizations, and families. The more established arrangements were the everyday support network within schools and the referral link between schools and the county medical system. The pseudo-professional mechanism emerged within this incomplete collaborative structure. Rather than waiting for every organization and professional post to be in place, the county embedded selected low-threshold functions in existing teacher-student relationships while using tiered referral to preserve professional boundaries. **Figure 1** summarizes the relationship between the three mechanism dimensions and the four stages of work.

### 4.1 Incomplete institutional collaboration and the emergence of a school-centered network

County policy documents established a leadership group, a guidance center, a training system, and a screening program. Interviewees nevertheless described the everyday participation of actors outside the education system as unstable. Medical services became involved mainly when high-risk cases were referred, while community organizations, local Women’s Federation offices, and social-service agencies had not become regular nodes in routine school support. In practice, school-family-community collaboration therefore took the form of a school-centered network, family participation, and school-hospital referral rather than sustained joint decision-making among several organizations.

This pattern does not mean that collaboration had failed altogether. Schools have regular access to students and stable organizational routines. Homeroom teachers, subject teachers, and school mental health teachers could translate policy requirements into everyday practices, while county-level initiatives supplied training, documentation, and referral procedures. Pseudo-professionalization developed as a transitional arrangement: before formal integration was complete, divisible support tasks were embedded in existing relationships and work routines.

### 4.2 Daily relational embeddedness: noticing risk in classroom and class life

Risk identification did not depend on standardized screening alone. It also relied on students being observed within continuing relationships. Some schools had developed an informal practice described by homeroom teachers as the “three daily observations”: facial expression, appetite, and general demeanor. These observations were incorporated into morning checks, meals, recess, and dismissal rather than organized as a separate diagnostic exercise.

“I insist that students eat breakfast before class, and if someone has not eaten, I ask why. I also eat lunch in the classroom. If a student eats far less than usual or looks completely listless, I notice immediately. We do not have to wait for a mental health class; the early signs appear in daily contact. One girl in my class barely touched lunch for two days. When I spoke with her in the corridor, she burst into tears. Several classmates had been talking about her online.” (homeroom teacher, urban junior secondary school)

Subject teachers formed a second observational node. County training materials encouraged teachers to attend to emotional and behavioral change while integrating age-appropriate mental health content into ordinary lessons. This orientation made students’ writing, drawings, classroom behavior, and peer interaction potential sources of warning information.

“The essay topic was ‘My worries.’ A boy who was usually very quiet wrote two full pages about his parents’ divorce and how neither of them paid much attention to him. He wrote as if life had no point. This was clearly more than an ordinary composition exercise, so I contacted his homeroom teacher immediately.” (Chinese-language teacher, township primary school)

Class mental health representatives and parent volunteers acted as supplementary nodes. After brief school-based training, the student representatives were expected to provide basic peer support, listen, and report concerns; they were not given lists of students under special attention, asked to assess risk, or permitted to manage high-risk situations alone. Peer proximity may reveal signals that teachers cannot see, but it also creates risks of labeling, disclosure, and excessive responsibility. Their role was therefore defined by prohibitions as well as assigned tasks: do not diagnose, do not hold sensitive records, and do not handle high-risk situations alone.

Students varied in how they experienced teachers’ attention. One junior secondary student valued that a teacher remembered a previous conversation about exam anxiety: “It felt as if someone had really heard me.” Another said that repeated questions created pressure when the student was merely tired or did not want to talk. Daily embeddedness made distress more visible, but support could still be experienced as surveillance.

### 4.3 Bounded task sharing: from listening and reassurance to risk reporting

Once a potential warning sign was noticed, the homeroom teacher commonly provided the first supportive response. The task was not to diagnose a condition. It was to create a reasonably safe opportunity to talk, listen to the student’s immediate concern, ask whether distress was persistent or worsening, and decide whether the case required prompt escalation. Some training converted professional knowledge into simple communication rules: listen for emotion, events, and needs; ask about feelings, expectations, and available support; do not judge, lecture, or rush to offer advice.

“If a student seems emotionally different, I will at least speak with them once. My first job is to listen. I need to understand whether this is a temporary response to a disappointing exam or something that has continued for a long time. The most important question is whether the student has thought about self-harm. That is the red line.” (homeroom teacher)

Extending the front end of support did not turn teachers into therapists. Interviewees repeatedly stated that teachers should not attach diagnostic labels or make clinical judgments. Clear self-harm marks, suicidal thoughts, psychotic symptoms, or other signs beyond the school’s competence had to be reported immediately to school mental health staff and administrators. School mental health teachers could assess risk and provide school-based support, but diagnosis and treatment remained the responsibility of qualified medical professionals.

“What we provide is initial reassurance and an understanding of the situation, not therapy. We cannot label a student and we cannot diagnose. If we see evidence of self-harm or hear a clear suicidal intention, the case must be reported immediately.” (homeroom teacher)

The safety of functional diffusion depended on training. The Education Bureau coordinated tiered training through the county guidance center, with contributions from external specialists. Lectures, case discussions, and simulations addressed common mental health problems, communication, warning signs, crisis procedures, and referral. Basic courses emphasized observation and initial reassurance; advanced courses covered risk assessment, case management, and crisis response. Mental health education was also incorporated into school supervision and evaluation. The system provided a foundation for bounded support work, but ongoing professional supervision and workload protection remained underdeveloped.

### 4.4 Tiered networking: professional intake, responsibility transfer, and parental consent

When a case exceeded the competence of homeroom or subject teachers, responsibility moved to the school level. School mental health teachers conducted school-based risk assessment, judged support needs, and provided supportive counseling. When a student showed clear self-harm or suicide risk, or signs of a serious mental disorder, the school contacted the guardian and initiated referral to medical services.

“When a homeroom teacher refers a student, I first conduct a school-based assessment. If the difficulty is an ordinary adjustment problem, I may arrange several brief support sessions. If there is severe anxiety, self-harm risk, or something such as hearing voices, it is clearly beyond the school’s capacity. We need to contact the family and refer the student to a hospital as soon as possible.” (school mental health teacher)

Case-management forms, responsibility lists, and a fast-track school-to-hospital referral pathway were intended to prevent referred cases from being left without an appropriate receiving service. The arrangement shortened waiting times for some families and allowed schools to obtain limited guidance for a student’s return.

“Before the fast-track referral pathway, we would advise parents to take a child to the hospital, but appointments could take one or two weeks. Some parents came back saying that the school was making a fuss, and then they simply did not go. Now the school contacts the hospital first and the family can attend at an agreed time, so the student can be seen more quickly.” (school mental health teacher)

The main bottleneck was often not the referral procedure but guardians’ understanding and consent. Some parents worried about diagnostic records, social labeling, and possible effects on schooling. A township homeroom teacher described the impasse plainly: “Some parents simply will not accept that the child may need help, let alone take the child to a doctor. We can complete every step on the school side and still reach an impasse with the family.” Tiered networking was therefore a process of relational negotiation as well as institutional transfer. Schools had to explain risk, treatment, and confidentiality in non-stigmatizing language. Information returned by medical services needed to be limited to what was necessary for school support and shared with the informed participation of guardians and students who were able to understand the decision.

### 4.5 Return-to-school support and everyday re-embedding

The support chain did not end when a student received medical care. Return to school required professional recommendations to be translated into everyday arrangements: temporary reduction of academic pressure, adjustment of assignments and activities, regular conversations, peer support, and two-way home-school communication. Homeroom and subject teachers again became the main actors, but their role was to support adaptation and notice change rather than to monitor treatment or obtain a complete medical history.

“A girl in my class took leave because of severe depression. When she returned after treatment, I spoke with all of her subject teachers. During the first week we did not check her homework, and if she could not follow a lesson, that was not the immediate priority. The first task was to help her readjust to school. I spoke with her for five minutes after class each day. When she went from keeping her head down and saying nothing to walking to the dining hall with classmates, I began to feel more reassured.” (homeroom teacher)

Parents valued coordinated support. One parent said that after the family reduced its emphasis on grades and began reporting changes in sleep, eating, and mood, “it no longer felt as if we were carrying the situation alone.” The continuity nevertheless depended heavily on teachers’ emotional labor. A township primary-school teacher described remaining anxious about saying something that might worsen a student’s condition.

A further weakness appeared at transitions between school levels. Warning information and effective support practices were often lost when students moved to a new school. A comprehensive psychological file, however, could cause a sensitive label to follow a student indefinitely. Continuity therefore should not mean unrestricted transfer of a complete history. Only information necessary for current safety and educational support should be shared, with informed participation, restricted access, and a defined retention period.

### 4.6 Internal tensions and points of breakdown

The pseudo-professional mechanism did not operate without cost. Relational proximity increased opportunities for early detection but also created additional emotional labor and responsibility for teachers. Short training could supply basic scripts without preparing staff for ambiguous, recurrent, or complex cases. Without ongoing supervision, teachers might over-report, delay reporting, or substitute moral instruction for listening. Parental consent remained a critical condition for medical referral and sustained support; stigma, cost, time, and fear of diagnostic consequences could interrupt the chain. Information sharing could improve continuity while widening exposure of sensitive data. Finally, weak participation by community and social-service organizations left gaps during holidays, outside school hours, and in families with limited caregiving capacity.

These tensions clarify the status of pseudo-professionalization. It is not a low-cost replacement for professional services and should not justify reducing investment in professional posts. Its defensible role is supplementary: early identification, low-threshold support, and regulated linkage while professional services continue to expand.

## 5 Discussion

The Suining case shows how a pseudo-professional mental health support mechanism can operate in a low-resource county. Where full-time professional coverage remained incomplete, selected front-end and peripheral functions were decomposed, translated into bounded tasks through training and procedures, and assigned to homeroom teachers, subject teachers, parent volunteers, and other actors within established relationships. The result was not a substitute for professional care, but a supplementary front-end network whose operation remained dependent on supervision, teacher capacity, parental participation, and referral resources.

### 5.1 From project-based task shifting to relationally embedded task sharing

The case shares a basic premise with international task-sharing research: professional services can be decomposed, and tasks can be allocated according to complexity and risk (12, 13). Its contextual contribution lies in the location of the shared tasks. They were not assigned primarily to newly recruited community health workers. They entered the existing occupational roles and interactional settings of school staff. Task sharing therefore activated relational resources as well as human resources.

This arrangement may lower the threshold for support and shift detection from occasional consultation to continuing contact. Existing relations, however, should not be equated with trust. Teachers hold evaluative and disciplinary authority, as well as authority over communication with families, and students may fear effects on school assessment or disclosure to parents. The value of relationally embedded task sharing must therefore be developed alongside confidentiality boundaries, student choice, and access to independent professional help.

### 5.2 From organizational integration to functional coordination

Collaborative governance research emphasizes face-to-face dialogue, trust, shared goals, and sustained commitment (24). The county case suggests that these conditions may not be simultaneously available at every stage of local service construction. Even without stable interdepartmental collaboration, limited coordination can form around particular functions: teachers notice and provide initial support, school mental health teachers assess risk and manage cases, medical institutions receive high-risk referrals, and families participate in treatment and return-to-school support. This pattern resembles the cross-actor linkage emphasized in accounts of integrated local governance, but its stability depends on professional nodes and institutional safeguards (25).

Functional coordination does not displace organizational integration. It can keep a basic support chain operating while formal collaboration remains incomplete, but it cannot replace community provision, clinical expansion, cross-sector responsibility systems, or public investment. The theoretical implication is therefore conditional: limited resources and incomplete integration do not necessarily stop all support, but supplementary functional reorganization remains viable only when minimum professional and institutional supports are present.

### 5.3 The meaning and conditions of pseudo-professionalization

Pseudo-professionalization refers here to a mechanism for organizing services, not to the identity of teachers or parents. Four conditions define its appropriate scope. Tasks must be divisible: only lower-risk, lower-threshold, supportive functions are suitable for sharing. Boundaries must be recognizable: actors need to know when to stop and refer. Professional nodes must be able to receive cases, provide feedback, and offer supervision. Relational embeddedness must remain subject to rights and privacy safeguards: everyday access cannot become unlimited monitoring or unrestricted information exchange. These conditions are consistent with task-sharing research that separates transferable from non-transferable service components and with the tiered service strategy of mhGAP (12–14).

The case extends Chinese research on adolescent mental health systems. Liu and Deng (26) emphasize institution-building from family to community, while Pei et al. (27) review multi-actor models linking schools, families, communities, government, and hospitals. Suining shows how school relationships may carry selected functions before full coordination is institutionalized. China’s “barefoot doctor” experience offers an analogy for extending access under scarcity (28), but different institutional, technical, and ethical conditions prevent historical comparison from establishing the safety of present practice.

### 5.4 Practical implications

County policy should not treat professional recruitment and non-specialist training as alternatives. Full-time posts, supervisory capacity among school mental health staff, and medical intake capacity remain the safety infrastructure of the system; task sharing extends the front end. Training should move beyond one-off lectures toward tiered curricula, simulation, regular supervision, and case consultation. Program design should also account for teachers’ time, provide emotional support, and protect staff from unreasonable liability.

Referral systems should specify risk levels, response timeframes, parent communication, emergency procedures, and return-to-school arrangements. Communication with families should avoid reducing psychological difficulty either to disease or to weakness of will and should explain risk and service options in accessible language. Information governance should follow the principles of purpose limitation and data minimization. Student mental health representatives, subject teachers without designated mental health responsibilities, and parent groups should not have access to lists of high-risk students or complete health records.

Further development of school-family-community collaboration must also strengthen support outside school. Community health services, social-work organizations, local Women’s Federation offices, and child-service agencies may contribute during holidays, in parent education, and in families with weak support capacity. Their participation, however, requires training, responsibility boundaries, and referral interfaces; a signed cooperation agreement alone does not constitute an operating collaborative system.

### 5.5 Limitations

This study concerns one county and can propose a mechanism without establishing universal applicability. Differences in professional resources, teachers’ work arrangements, medical access, and the density of local social ties may alter how the mechanism operates. Participants were recruited through schools and the education system; students who had left school, families that declined referral or treatment, and young people outside the support network are underrepresented. The student sample was small and did not cover all grades or school types. The administrative platform lacked complete psychometric documentation available for public verification, so its risk flags are contextual information only. The study did not collect mental health outcomes, service-use measures, false-positive rates, costs, or longitudinal follow-up and therefore cannot determine clinical effectiveness or cost-effectiveness. Finally, access through education authorities and schools may have encouraged favorable accounts of institutional practice. Comparative county studies, independent recruitment, and longitudinal research are needed.

## 6 Conclusion

The Suining case indicates that adolescent mental health support in a resource-constrained county need not be postponed entirely until professional and cross-sector capacity is fully developed. General observation, supportive listening, basic reassurance, and connection to services can be placed within the everyday relationships of homeroom teachers, subject teachers, and families, while school mental health teachers and medical institutions retain professional gatekeeping and referral responsibilities.

The value of this mechanism lies in widening access to front-end support, not in demonstrating that non-professional services can replace clinical care. Its safety and continuity depend on professional investment, sustained training and supervision, clear referral thresholds, informed participation by students and families, protection of teachers’ time and capacity, and disciplined information governance. The relationally embedded task-sharing framework derived from this single community case provides a preliminary explanation of how low-resource counties may organize support between policy ambition and limited service capacity. Its boundary conditions require comparative and longitudinal examination.

## Data Availability

The raw data supporting the conclusions of this article will be made available by the authors, without undue reservation.
